# A Combined Approach to Infrared Small-Target Detection with the Alternating Direction Method of Multipliers and an Improved Top-Hat Transformation

**DOI:** 10.3390/s22197327

**Published:** 2022-09-27

**Authors:** Tengyan Xi, Lihua Yuan, Quanbin Sun

**Affiliations:** 1 Key Laboratory of Nondestructive Testing (Ministry of Education), Nanchang Hang Kong University, Nanchang 330031, China; 2 School of Computing and Digital Technology, Birmingham City University, Birmingham B5 5JU, UK

**Keywords:** infrared image, small-target detection, alternating direction method of multipliers, new top-hat, signal to clutter ratio, background suppression factor

## Abstract

In infrared small target detection, the infrared patch image (IPI)-model-based methods produce better results than other popular approaches (such as max-mean, top-hat, and human visual system) but in some extreme cases it suffers from long processing times and inconsistent performance. In order to overcome these issues, we propose a novel approach of dividing the traditional target detection process into two steps: suppression of background noise and elimination of clutter. The workflow consists of four steps: after importing the images, the second step applies the alternating direction multiplier method to preliminarily remove the background. Comparatively to the IPI model, this step does not require sliding patches, resulting in a significant reduction in processing time. To eliminate residual noise and clutter, the interim results from morphological filtering are then processed in step 3 through an improved new top-hat transformation, using a threefold structuring element. The final step is thresholding segmentation, which uses an adaptive threshold algorithm. Compared with IPI and the new top-hat methods, as well as some other widely used methods, our approach was able to detect infrared targets more efficiently (90% less computational time) and consistently (no sudden performance drop).

## 1. Introduction

Infrared small-target detection is a key technology in infrared search-and-track systems and has been widely used in many areas, such as aerial reconnaissance, early warning, military surveillance, and reconnaissance [1,2,3], for its advantages of long-range detection, full-time operation, and anti-interference. However, due to the nature of light scattering and energy loss in long-distance imaging, the targets are captured in small sizes and often with clutter and noise, resulting in images with low signal-to-noise ratios [4,5,6]. Despite many efforts made in the past decades (as outlined below), the detection of small infrared targets remains a challenge, especially in an environment of complex backgrounds or when detecting extra-small targets.

Generally, infrared small-target detection methods fall into two categories: multiframe image detection, and single-frame image detection. Multiframe image detection utilizes the prior information of the target and background from the previous frame(s) to detect the moving targets. While early studies/algorithms were mostly implemented through dynamic programming [7], three-dimensional matched filtering [8], and multilevel hypothesis testing [9], studies after the 2000s often used combined methods, e.g., using a direction-matched filter based on spatiotemporal information to suppress clutter [10], or a combination of a bilateral filter and a temporal cross product (BF-TCP) [11] with temporal cross-entropy to generate spatiotemporal images to detect targets. In recent years, methods such as the Markov random field guide noise model [12], spatial-temporal local contrast filtering (STLCF) [13], spatial-temporal features measure (STFM) [14], or guided filtering and convolution neural networks were used in many studies [15]. However, those approaches often required prior information about the image and suffered from high time complexities, which was due to numerous calculations on the motion trajectories of all potential targets to determine the potential targets. Furthermore, their performances still depended on the detection performance of a single frame image, which therefore limited their applications in practice. As a result, more studies now focus on the single-frame image approach [16].

Early studies on single-frame images aimed to improve the contrast and signal-to-noise ratio by enhancing targets and suppressing noise, either through linear or nonlinear filtering, or by estimating the background component with preprocessing. Popular algorithms include max-median, max-mean [17], and wavelet transformation [18], which work well when the background in the image is simple. However, significant change in the background, where the grey value gradient of the corresponding area changes greatly, often results in a false detection. In addition, the complex backgrounds also cause high false alarm rates for similar reasons. To tackle such issues, some studies took a morphological approach. In morphology, the scale of the structuring element is a critical parameter; its value determines the accuracy of feature extraction [19] by utilizing the prior knowledge of the size and shape of the target to construct the appropriate structuring elements and then obtain the detection image through the differential operation between the original image and the filtered image, such as in traditional top-hat [20] and adaptive top-hat methods [21,22]. The structuring element has a great impact on the top-hat operation and the new white top-hat transformation (NWTH) [23] is often considered the best in the morphological approach, along with some improved ring top-hat transformation [24,25,26].

Alternatively, human visual system (HVM) was introduced to infrared small-target detection in 2013 when the local contrast measure (LCM) method [27] was proposed. This approach considers the target, and the background can be separated by constructing different local contrasts, as the grey values of the small target are higher than the grey values of the background. It provided both initial good detection performance and short processing time, hence many further studies were carried out, such as improved LCM (ILCM) [28], relative LCM (RLCM) [29], and novel LCM (NLCM) [30]. In addition, the method combined with other modules, such as the method of combining weighted double-layer local contrast and multidirectional map [31], which realizes the detection of small targets in terms of local contrast and gradient of targets, and the method of combining the Laplace of Gaussian (LoG) filter and the negative LoG filter with local contrast [32]. However, such methods show mediocre effects in background suppression when dealing with bright backgrounds and they are also known for the corresponding phenomenon of noise point enhancement.

Based on the characteristics of the infrared small target image, Denney et al. [33] first suggested the target detection problem could be converted into a robust principal component analysis (RPCA) problem. Gao et al. [34] further proposed an infrared patch image model (IPI) that considered the infrared background as a single low-rank subspace and therefore the small target should be regarded as an outlier, i.e., the small-target detection problem became an optimization problem of low-rank-matrix and sparse-matrix recovery. In IPI, the infrared image was first processed and transformed into smaller image patches. Then, the accelerated proximal gradient (APG) algorithm was used to solve the low-rank- and sparse-matrix separation problem. Such an approach showed superior results but required a significantly longer processing time, normally in seconds or tens of seconds (for comparison, the processing times of the methods based on the aforementioned approaches were under 0.1 s). In addition, its performance could be affected by serious clutter and noise. To address such issues, some studies considered replacing APG with the alternating direction method of multipliers (ADMM), such as the weighted IPI model (WIPI) [35] and the non-negative IPI model, by minimizing the partial sum of singular values (NIPPS) [36]. Despite the excellent detection performance, the high time complexity of this approach is yet to be resolved.

With the development of deep learning in the field of computer vision, some studies applied this method to infrared small-target detection [37,38,39,40]. Such an approach provides comparative performance but requires training the model with a large amount of data in advance. Furthermore, such models rely on the types of training data, i.e., the detection performances on various and new backgrounds/scenarios may vary. Although the deep learning model and network structure are becoming lighter and lighter [41,42], it is still a challenge to apply these models to the field of infrared small-target detection because the characteristics of the infrared small target only occupy individual pixels and fuzzy textures.

To address such issues, we propose a novel combined approach to further improve the excellent detection performance of IPI models, as well as to significantly reduce the processing time by incorporating an improved NWTH transformation with a specifically designed threefold structuring element. Our method produced a consistent performance across all five testing image sequences. It used 90% less time than the IPI method and scored the best in all metrics overall as compared to the methods using a single approach.

The paper is organized as follows: Section 2 presents the RPCA and morphological approaches and their recent developments which underpin our study. Section 3 describes the combined approach and the workflow of the proposed method. Section 4 shows the experimental results and the evaluation against six state-of-art methods. The conclusion is presented in Section 5.

## 2. Related Work

### 2.1. Robust Principal Component Analysis (RPCA)

**Figure 1:**
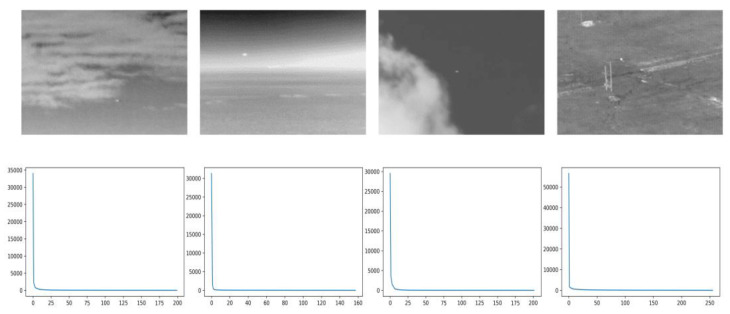
Infrared image and its corresponding singular value curve.

In infrared small-target detection, on one hand, the infrared images normally have a characteristic of nonlocal autocorrelation [43]; thus, the background can be represented by a low-rank matrix. For example, Figure 1 shows four classic infrared images and their corresponding singular value curves. Although the images have different backgrounds, their singular values converge to zero quickly.

On the other hand, the small target can be considered a sparse matrix when the target area is less than 15% of the total image and the signal-to-clutter ratio between the target and the background is less than 4 dB [27]. As a result, the original infrared image is composited of a background image with low-rank characteristics and a foreground image containing a small target showing sparse characteristics and noise. Therefore, RPCA can be used to separate the background and the foreground.

### 2.2. Alternating Direction Method of Multipliers (ADMM)

The ADMM algorithm is a method to solve the RPCA convex optimization problem [35,36] through which the infrared image is taken as the low-rank data observation matrix D. However, when D is affected by random noise, its low-rank characteristic disappears, and *D* becomes full rank. One solution is to convert the constrained optimization into an unconstrained optimization through convex optimization, i.e., to decompose D into a low-rank matrix and a sparse matrix to its real structure. As a result, the RPCA problem can be represented in the following form:

(1) $$\underset{A,E}{\min}{∥A∥}_{*}\;+\;\lambda {∥E∥}_{1}\;+\;\gamma {∥N∥}_{F}^{2}\;\;\;s.t.D\;=\;A\;+\;E\;+\;N$$

where ${∥A∥}_{*}\;=\;\sum_{i=1}^{m}\delta_{i}\left(A\right)$ represents the kernel norm of matrix A; $\delta_{i}\left(A\right)$ represents the ith singular value of A; λ is the weighting of the noise; ${∥E∥}_{1}\;=\;\underset{ij}{\sum }\left|E_{ij}\right|$ represents the sum of the absolute values of all elements in matrix E; A is the background; E is the target; N is the random noise; and γ is the weighting of the random noise with a small value. The augmented Lagrange function of Equation (1) is defined as follows:

(2) $$L_{\rho }\left(A,E,N,G,\rho \right)={∥A∥}_{*}\;+\;\lambda {∥E∥}_{1}\;+\;\gamma {∥N∥}_{F}^{2}\;+\;\langle G,D-A-E-N\rangle \;+\;\rho /2∥D-A-E-{N∥}_{F}^{2}$$

where ρ is the penalty factor; G is the Lagrange multiplier; and $\rho /2∥D-A-E-{N∥}_{F}^{2}$ represents the square regular term, as the additional constraint when compared to Equation (1).

### 2.3. Top-Hat and NWTH

In the traditional top-hat method [20], separated the target by subtracting the result of the original image after the opening operation from the original image. In morphology, opening is used to eliminate bright pixels (i.e., the target) from an infrared image via a carefully constructed structuring element. The opening operation is defined as follows:

(3) f(x,y)∘B=(f(x,y)⊖B)⊕B

where f(x,y) represents the original image; B is the structuring element; ∘ represents the opening operation; ⊕ is the dilation operation; and ⊖ is the erosion operation.

**Figure 2:**
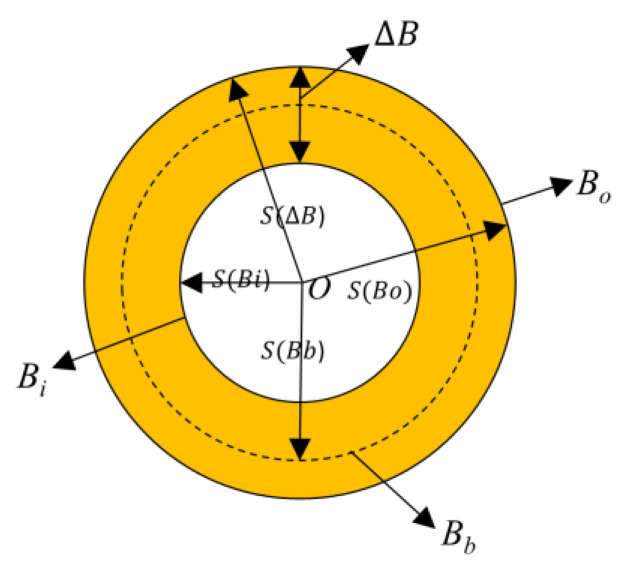
Relationship of the structuring elements in NWTH [23]. ΔB consists of $B_{i}$ and $B_{o}$, ΔB and represent structural elements for dilation and erosion operations, respectively.

To better tackle the heavy clutter and noise, the new white top-hat method (NWTH) [23] proposed a new operation by swapping the order of erosion and dilation as below:

(4) $$f\left(x,y\right)■B_{oi}=\left(f\left(x,y\right)⊕\Delta B\right)⊖B_{b}$$

where ■ represents new operation; and ΔB and $B_{b}$ represent structural elements in Figure 2. In addition, a different but correlated structuring element (see Figure 2) were used for both erosion and dilation.

## 3. The Proposed Method

### 3.1. Overview

On one hand, although the RPCA-based IPI methods generally produced better results, they suffered from long processing times, which, in most cases, were among tens of seconds. This was due to the nature of reconstructing the matrix via the patch images using the nonlocal autocorrelation and cannot be easily improved without fundamentally changing the algorithms. On the other hand, methods using the morphologic or the HVM approaches could run much faster (under one second) and were still able to produce good results. Our motivation was to explore whether a combined approach could be made possible to achieve both a better result and a short processing time.

For a given infrared image, it can be decomposed as target, background, and noise [34]:

(5) $$f_{D}\left(x,y\right)=f_{T}\left(x,y\right)+f_{B}\left(x,y\right)+f_{N}\left(x,y\right)$$

where (x,y) is the coordinate of the pixel; $f_{D}$ is the original infrared image; $f_{T}$ represents the target; $f_{B}$ represents the background; and $f_{N}$ represents the noise.

**Figure 3:**
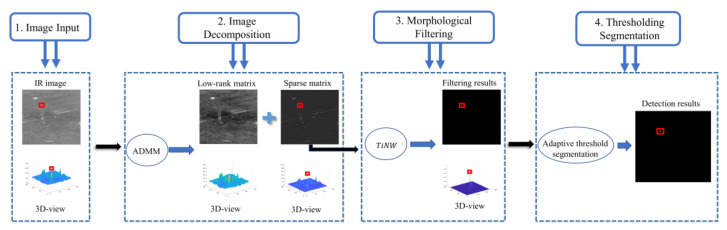
The workflow of the proposed method. red squares represents small target.

Rather than using a single step to separate the target matrix $f_{T}$ directly, we proposed to first separate the background $f_{B}$ from the image, which can be described as a typical RPCA problem (where ADMM could be used without using IPI). In the next step, with the new image having most of the background removed, the morphological filtering method could be performed. We considered NWTH to be a good choice, as it was specifically designed to tackle noise and clutter. It is worth pointing out that although the morphological approach was not good at dealing with the complex background, such a weakness would not be exposed in our combined approach wherein the background was already preliminarily removed in the first step. We did not choose the HVM-based approach because it had issues dealing with images that had bright backgrounds where such a character could not be mitigated in the first step. As a result, our proposed method is described in Figure 3.

### 3.2. Image Decomposition—ADMM

The goal of this step was to separate the background from the image, which was an RPCA-related problem as discussed in Section 2. As compared to the traditional RPCA-based methods wherein both the background and the noise were suppressed in a single process, in our approach, the image decomposition step had a much higher tolerance and allowed partially residual background and noise, which would be suppressed at the next step of morphological filtering, i.e., the decomposition aimed to mainly separate $f_{B}$ from Equation (1). Therefore, this step was considered a preliminary suppression and ADMM could be applied to the whole image directly. As compared to the IPI-based methods, our approach did use process patch images with a sliding window, and thus could significantly improve the processing time.

To attack the problem defined by Equation (2), only one of A, E, and G was targeted to solve the proximity function at each iteration, while the other two were fixed. The pseudocode of the algorithm is shown in Algorithm 1.
**Algorithm 1** The pseudocode of ADMM.**Output: Sparse matrix**$A_{k}$**and low-rank matrix**$E_{k}$**let**$\lambda =1/\sqrt{max\left(m,n\right)},E_{0}=0,N_{0}=0,k=1,\sigma =2$; **while** (not converged)       
$\left(U_{k},\;\sum_{k},\;V_{k}\right)=SVD\left(D-E_{k}-N_{k}+\rho_{k}^{-1}G_{k}\right)$
       $A_{k+1}=U_{k}S_{\rho_{k}^{-1}}\left(\sum_{k}\right)V_{k}^{T}$;       $E_{k+1}=S_{\lambda /\rho }\left(D-A_{k+1}-N_{k}+\rho^{-1}G_{k}\right)$;       $N_{k+1}=\rho /\rho +2\gamma \left(D-A_{k+1}-N_{k+1}+\rho^{-1}G_{k}\right)$;       $G_{k+1}=G_{k}+\rho_{k}\left(D-A_{k+1}-E_{k+1}-N_{k+1}\right)$;       $\rho_{k+1}=\sigma \rho_{k}$;       k=k+1; **end**

k represents the number of iterations; σ represents the coefficient of the penalty factor at each iteration; $SVD\left(D-E_{k}-N_{k}+\rho_{k}^{-1}G_{k}\right)$ represents the singular value decomposition of matrix $D-E_{k}-N_{k}+\rho_{k}^{-1}G_{k}$; $U_{k}$ and $V_{k}$ represent the left and right orthogonal matrices of the singular value decomposition of matrix $D-E_{k}-N_{k}+\rho_{k}^{-1}G_{k}$; $\sum_{k}$ represents the diagonal matrix composed of the eigenvalues of the singular value decomposition; $S_{\rho_{k}^{-1}}$ represents the contraction operator given the specific penalty factor $\rho_{k}^{-1}$.

After decomposing the low-rank matrix (i.e., the background $f_{B}$), the remaining components of the image consisted of the target $f_{T}$ and the noise, which included the original noise $f_{N}$ plus the residual background.

### 3.3. Morphological Filtering—An Improved NTWH Transformation

**Figure 4:**
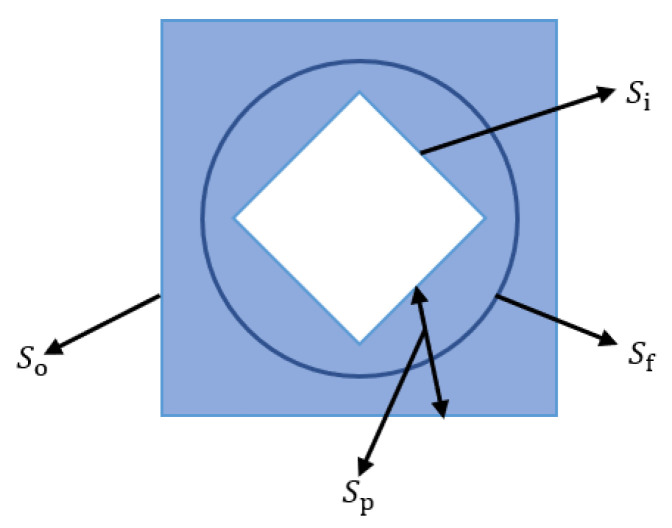
Relationship of the threefold structuring elements in our improved NWTH transformation.$\;S_{p}$ consists of $S_{i}$ and $S_{o}$, $S_{p}$ and $S_{f}$ represent structural elements for dilation and erosion operations, respectively.

**Figure 5:**
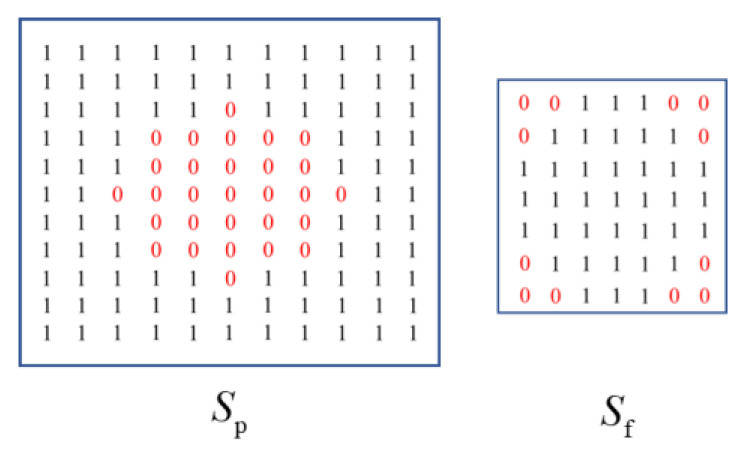
The matrix representations of the structuring elements.

To better identify the small targets from the decomposed sparse-matrix image, we constructed a threefold structuring element when adapting the top-hat-based method NWTH [23]. The structuring element is shown in Figure 4. $S_{p}$ is the structuring element for dilation, which is formed by $S_{i}$ subtracting $S_{o}$. $S_{o}$ (a square) represents the outer shape of $S_{p}$, for which the size is slightly larger than the target, while $S_{i}$ (a square diamond) represents the inner shape of $S_{p}$, for which the size is slightly smaller than the target. $S_{f}$ (a circle) is the structuring element for erosion, for which the size should be between $S_{i}$ and $S_{o}$. The matrices of $S_{p}$ and $S_{f}$ are shown in Figure 5, where “1” represents the structuring elements.

**Figure 6:**
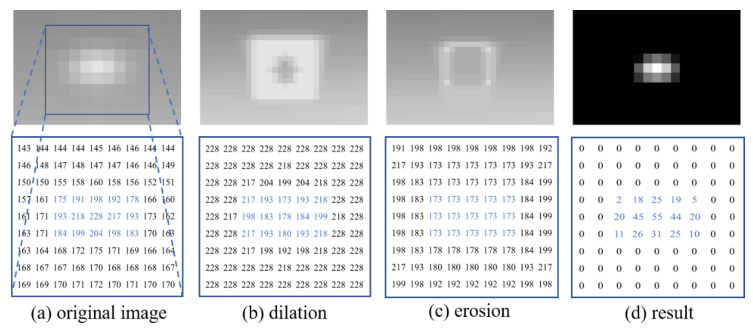
The process of morphological filtering: (**a**) Small target area in the original image; (**b**) dilation; (**c**) erosion; (**d**) the results of our improved NWTH. A 9 × 9 matrix of the target region is shown at each step.

To demonstrate how this threefold structuring element works, the process of the improved NWTH transformation is shown in Figure 6, wherein the images of the target region at each step are at the top and their corresponding matrices are at the bottom. The target is in the shape of 5 × 3, and its corresponding pixels are highlighted in blue in Figure 6a. With the specifically constructed structuring element $S_{p}$, the pixels in the surrounding area of the target all gained the local maximum values via dilation. Figure 6b shows the target was successfully highlighted by a rectangle (of bright pixels) while the target itself was restrained in a smaller diamond shape (of grey pixels). The result of erosion is shown in Figure 6c, wherein the target was enlarged into a rectangle (of grey pixels) with a highlighted outer boundary (of bright pixels). This was because of the circle structuring element $S_{f}$ had a size between the outer boundary and the inner boundary of $S_{p}$. The final result was obtained by subtracting Figure 6c from Figure 6a, wherein all background and noise were eliminated and the target was successfully obtained, as shown in Figure 6d. During the substruction, the pixels in the nontarget regions might result in negative values, which should be set to 0. As a result, our improved NWTH transformation was defined as below:

(6) $$TiNW=f\left(x,y\right)-min\left(\left(f\left(x,y\right)⊕S_{p}\right)⊖S_{f},\;f\left(x,y\right)\right)$$

where f(x,y) represents the original image; *min* is the minimum operation; ⊕ is dilation; ⊖ is erosion; and $S_{p}$ and $S_{f}$ are defined in Figure 4. To better eliminate the clutter and noise in the target region, the sizes of $S_{i}$ and $S_{o}$ for $S_{p}$ should be adjusted accordingly.

### 3.4. Adaptive Thresholding Segmentation

After the above steps, there might be a few false alarm points which generally occupied only one or two pixels. To eliminate them, the image was binarized via adaptive thresholding segmentation. Threshold ***T*** was defined as [24,44]:

(7) T=M+k×S

where M and S represent the average value and standard deviation of the image after background suppression, respectively, and k is an empirical constant which has its value set to 40 in this study.

## 4. Experimental Results and Analysis

### 4.1. Experimental Setup

#### 4.1.1. Hardware and Software

The simulation experiment was carried out in a MATLAB r2020b environment. The experimental hardware used an HPS-P18C32GB workstation, with Intel Xeon scalable platinum 8124 m 3.0 GHz, and 32 GB DDR RAM.

#### 4.1.2. Datasets

To test and verify the performance of the proposed method, five sequences of images were selected from open-sourced infrared image datasets [45,46] and their properties are shown in Table 1.

#### 4.1.3. Baseline Methods

Six methods were selected for comparison, including the traditional max-mean algorithm [17] as a baseline method, two morphological filtering methods (the classical top-hat transform [20] and the new top-hat method NWTH [23]), two HVS-based methods (the LCM algorithm [27] and the RLCM algorithm [29]), and one RPCA-based model (IPI [34]). The parameters of such methods are shown in Table 2.

#### 4.1.4. Evaluation Metrics

To quantitatively measure the effectiveness of target highlighting and background compression, signal-to-clutter ratio (SCR) and background suppression factor (BSF) [12,13,34,35] are the two commonly used metrics:

(8) $$SCR=\left|\mu_{t}-\mu_{b}\right|/\sigma_{b}$$

(9) $$BSF=\sigma_{in}/\sigma_{out}$$

where $\mu_{t}$ represents the mean values of target pixels; $\mu_{b}$ represents the mean of the background pixels around the target; $\sigma_{b}$ represents the standard deviation of that background; and $\sigma_{out}$ and $\sigma_{in}$ represent the standard deviation between the output image and the input image. BSF and SCR are calculated against the images before thresholding segmentation. The larger the value, the better the target detection and background compression effects of the algorithm.

False alarm rate ($F_{a}$) [34,46,47] is used to describe an algorithm’s capacity for making correct detections. The calculation uses the result after thresholding segmentation. In this paper, we adopted the definition of the falsely detected pixels:

(10) $$F_{a}=N_{f}/N_{w}$$

where $N_{f}$ represents the number of pixels that are falsely detected and $N_{w}$ represents the total number of pixels of the whole image.

To evaluate an algorithm’s effect on a sequence of infrared frames, the average values ($\bar{SCR}$, $\bar{BSF}$, and $\bar{F_{a}}$) are defined as below:

(11) $$\bar{SCR}=\frac{1}{N}\sum_{i=1}^{N}SCR_{i}$$

(12) $$\bar{BSF}=\frac{1}{N}\sum_{i=1}^{N}BSF_{i}$$

(13) $$\bar{F_{a}}=\frac{1}{N}\sum_{i=1}^{N}F_{a}{}_{i}$$

where N represents the total number of frames in the sequence; $SCR_{i}$, $BSF_{i}$, and $F_{a}{}_{i}$ represent the values of SCR, BSF, and $F_{a}$ of the ith frame.

The processing time of each frame was recorded to calculate the average processing time of each image sequence.

### 4.2. Experimental Results: Results at Each Stage, in Four Typical Backgrounds

**Figure 7:**
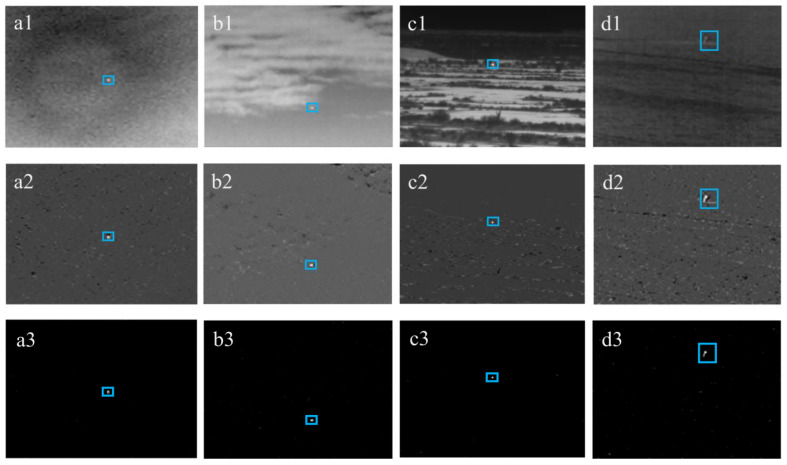

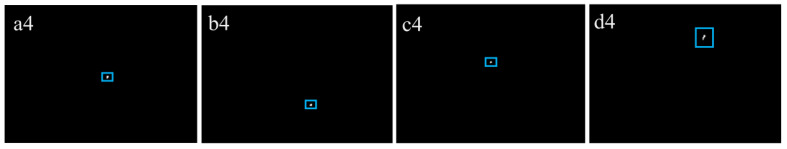
The simulation results at each stage of four typical backgrounds: (**a1**) sky, (**b1**) cloud, (**c1**) land, and (**d1**) sea; (**a2**–**d2**) show low-rank-matrix image after image decomposition; (**a3**–**d3**) displays the images after the morphological filtering stage; (**a4**–**d4**) show final results.

To better illustrate the workflow of the proposed method, four typical backgrounds, i.e., (a) sky, (b) cloud, (c) land, and (d) sea, were selected from the SIRST dataset [48]. The simulation results of each of the four steps are shown in Figure 7. Row one represents the original infrared images, respectively, wherein the targets are circled with a blue rectangle. Row two shows the low-rank-matrix image after image decomposition (i.e., ADMM), wherein the background was preliminarily suppressed. Row three displays the images after the morphological filtering stage (i.e., the improved top-hat transformation), wherein the targets were successfully separated. At this stage, the background suppression was completed and most of the background noise was eliminated. While scatters of noise might still exist, they were further removed at the adaptive threshold segmentation step. The results are shown in row four, wherein the targets were identified with no concern for false alarm points.

**Figure 8:**
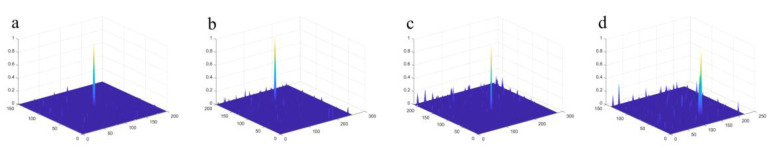
Three-dimensional views of the improved Top-hat operation results: (**a**) sky, (**b**) cloud, (**c**) land, and (**d**) sea.

Figure 8 shows the images after the morphological filtering step in three-dimensional diagrams, in which the backgrounds were effectively suppressed. In all images, there was no false alarm point observed.

### 4.3. Experimental Results: Comparison to the State-of-Art Algorithms

To evaluate the effectiveness and adaptability of the proposed method, the comparisons were carried out in three aspects: visual observation, quantitative measurement, and overall performance.

#### 4.3.1. Visual Observation

**Figure 9:**
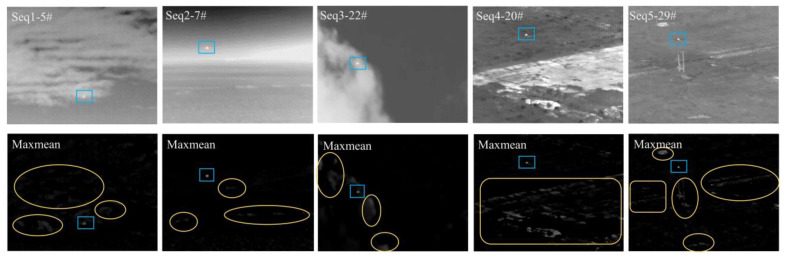

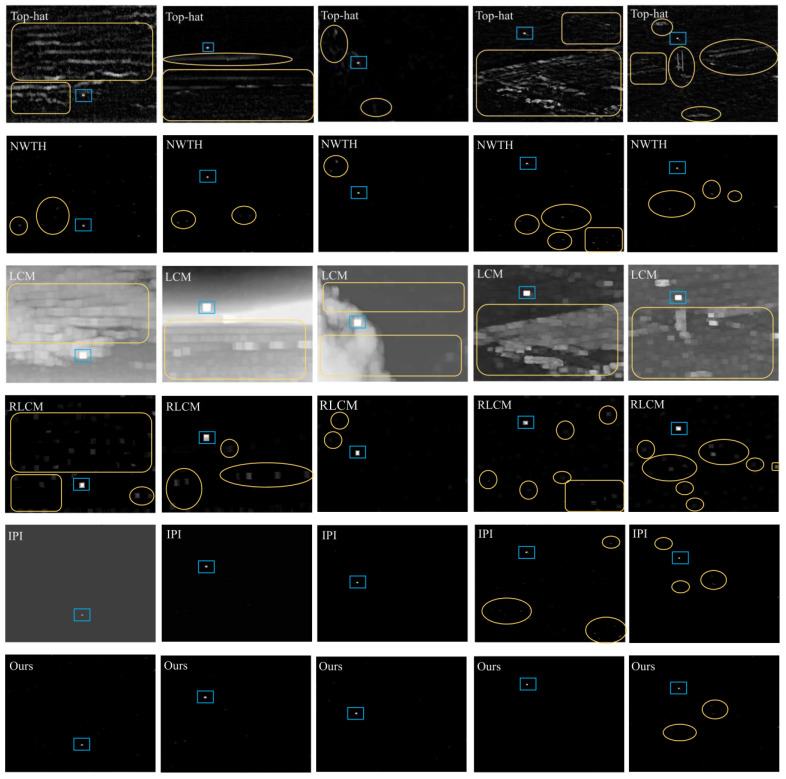
Background suppression results of the six methods (Table 2). # indicates the frame number. The blue rectangle represents the target, while the yellow box shows the false alarm point or the unsuppressed background.

The visual comparisons are shown in Figure 9 (the results before the adaptive threshold segmentation step were used). In terms of background suppression, the effect of max-mean was mediocre and most of the high-frequency background remained in all five sequences. For the classic top-hat and the LCM methods, both effects were insufficient as a large amount of continuous background clutter was left in every image. The RLCM method produced much better results as compared to the three. However, due to the nature of contrast enhancement, some noise points became more prominent, which were observed in sequences 1, 2, and 5. For NWTH, IPI, and our method, the background suppression effects were excellent.

In terms of detecting the correct targets, all six methods were able to highlight the target regions, despite many also falsely highlighting noise points (however, those noise points did not necessarily become false alarm points after adaptive threshold segmentation). For the max-mean, the traditional top-hat, and both LCM methods, their detecting capacities were restricted by their background suppression effects, i.e., the residual bright background would mostly result in false alarm points. The NWTH method had bright points left in all five sequences, which indicated the structuring element of the top-hat transformation could be refined for the selected image sequences with complex backgrounds. The IPI method produced the second-best outcomes with some potential false detections in sequences 4 and 5 which had fast-changing backgrounds with a tracking camera position. Our method had the best visual representations with two minor noise points in sequence 5.

**Figure 10:**
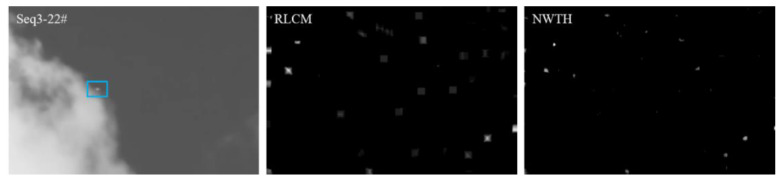
No target was detected by RLCM and NWTH in sequence 3 in some frames.

Figure 9 shows the most representative frame from each image sequence, aiming to demonstrate the method’s overall performance on the given sequence. However, it is worth pointing out that some methods did perform inconsistently on certain frames, which resulted in a false alarm or nondetection. For example, the NWTH method did not detect any target from frames 11 to 13 in sequence 3 (Figure 10); the RLCM method was not able to detect any target from frames 11 to 15 in sequence 3 (Figure 10); the IPI method retained the background components in nearly half of the frames (i.e., 1–6, 10–12, and 16) in sequence 1 (see Figure 9, column 1, row 7 for example), which resulted in low SCR values. Since the image datasets did not provide any timestamp on each frame, we were not able to identify the real time elapsed in those frames. Therefore, we could not evaluate to what extent those inconsistent performances would affect the accuracy of monitoring or surveillance in the real world.

#### 4.3.2. Quantitative Comparison

The results of the average SCR are shown in Table 3. Our method had three best scores (seq 1, 2, and 4) and two second-best scores (seq 3, 5), which demonstrates that our method was superior in highlighting the target. NWTH, RLCM, and IPI had similar overall rankings, which were considerably better than max-mean, top-hat, and LCM. More specifically, our method produced much better SCR values (240+) than all methods in sequences 1 and 2. In sequence 3, the LCM had the best SCR value. Although our score (490.00) came second, it was considered sufficient and showed a clear advantage over both the NWTH and IPI results. In sequence 4, our method scored the best (35.26), while most other methods had values around 10. In sequence 5, the IPI had a distinct SCR value over others, but our result (20.68) was still much larger than the results (around 10) of the remaining five methods.

The results of the average BSF are shown in Table 4. NWTH, IPI, and our method had much better values than max-mean, top-hat, LCM, and RLCM in all five sequences, while IPI and our method showed superior results to NWTH, especially in sequences 1 and 4. As compared to the IPI algorithm, our method scored one best (seq 3) and four second-best (seq 1, 2, 4, and 5). Such results were considered on par with IPI (four best and one second best) as the differences in sequence 2 and sequence 5 were merely 2%.

For the processing time, the results of three methods (NWTH, IPI, and our method) are shown in Table 5. NWTH was an algorithm using the morphological approach that had a very short processing time due to the simplicity of matrix operations. It yielded the best processing times in milliseconds, with an average time of 0.016 s across all five sequences. IPI was an algorithm adapting RPCA, which required a significant amount of processing time due to the complexity of the sliding steps. It had the worst processing time among the three, with an average of 29.28 s. Although our method combined both the RPCA and morphological approach, it did not require a small patch in image decomposition, i.e., it had a much smaller time complexity than IPI. Our method had an average processing time of 2.25 s, which was a huge improvement (92% less) from IPI and could be considered as near-real-time detection.

**Figure 11:**
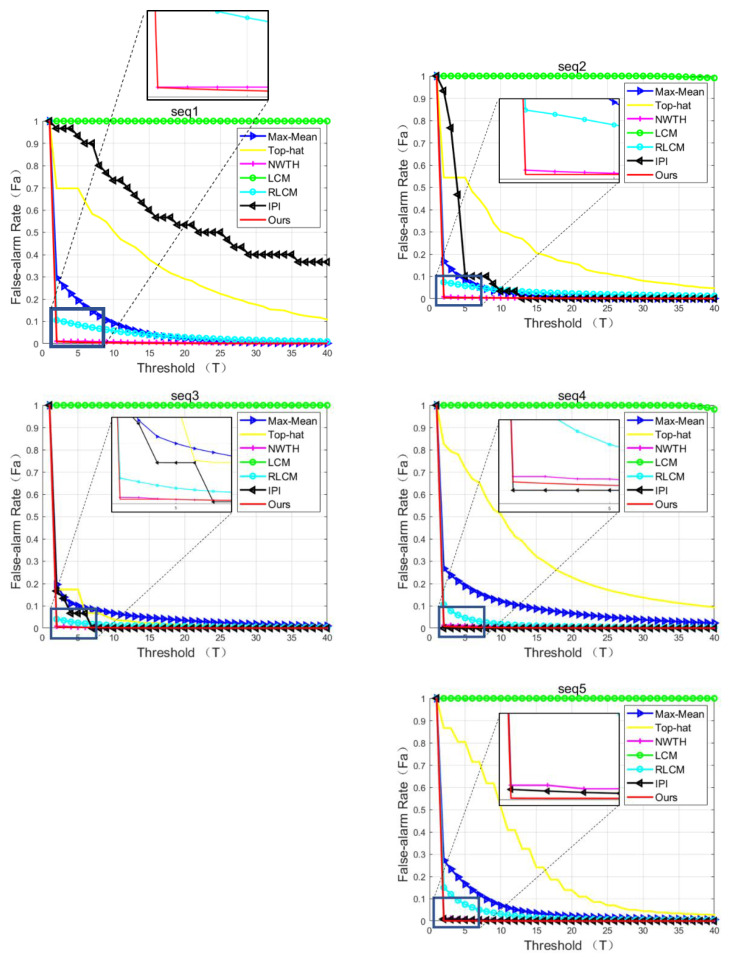
False alarm rates over threshold levels. The *Fa* represents the average of all 30 frames of that sequence.

The false alarm rates over threshold levels are shown in Figure 11, which indicated that NWTH, IPI, and our method were superior to max-mean, top-hat, LCM, and RLCM in all sequences (except IPI in sequence 1). More specifically, when compared to NWTH, our method achieved negligible better false alarm rates in sequences 1–3 but clear better results in sequences 4 and 5, in which the background became more complex and/or the targets were smaller. When compared to IPI, our method had better rates in sequences 1–3, and 5, while IPI was better in sequence 4. It is worth pointing out that the IPI method showed poor false alarm rates at low thresholds in sequences 1–3 and such results were considered due to its inconsistent background suppression performance, as discussed in the previous visual observation section.

#### 4.3.3. Overall Comparison

Based on the comparisons above, all NWTH, IPI, and our method exhibited a clear superior performance to max-mean, top-hat, LCM, and RLCM in both visual observations and quantitative (SCR, BSF, and $F_{a}$) comparisons. Despite showing clutter and noise in the visual observation, NWTH was able to eliminate most of them via threshold segmentation, thus achieving much better false alarm rates than the other four algorithms. However, NWTH was inferior to both IPI and our method in almost every aspect except for processing time. When compared to IPI, our method showed better visual and SCR results and similar results in BSF, which might suggest that both methods possess similar target detection performances. However, the IPI method showed poor $F_{a}$ rates at low thresholds in sequences 1–3 due to its unstable background suppression effects in certain situations. In addition, the main drawback of IPI is the high processing times required, whereas our method had a clear advantage, using approximately 8% of IPI’s processing time on average. In a nutshell, our method showed the best overall performance and had wider applications than IPI thanks to its relatively low processing time (2.25 s on average).

### 4.4. Additional Experimental Results: Effectiveness of Our New Top-Hat Structuring Element

**Figure 12:**
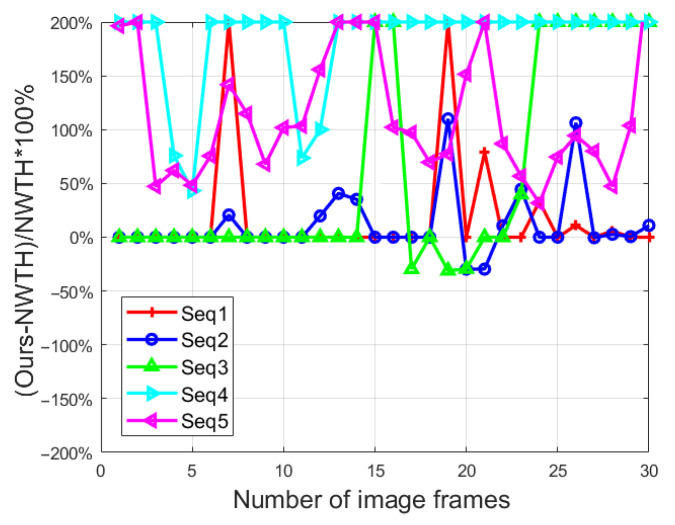
The ratio of SCR difference to NWTH. Capped at 200% for better presentation.

**Figure 13:**
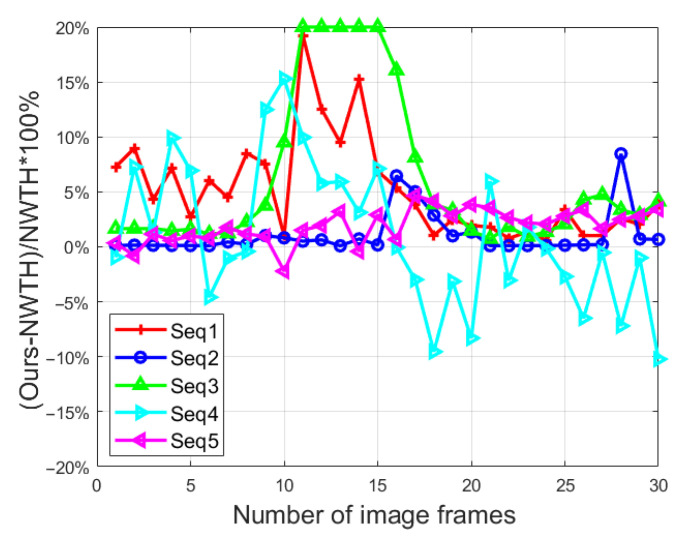
The ratio of BSF difference to NWTH. Capped at 20% for better presentation.

In the morphologic filtering step of our method, we proposed a threefold structuring element (see Figure 4) as an improvement to NWTH. To further evaluate its effectiveness, an additional experiment was run using the improved transformation TiNW (see Equation (6)) on all five sequences in which both BSF and SCR were captured. Figure 12 and Figure 13 showed the ratios of the differences between our improved transformation and the original NWTH for both SCR and BSF.

For SCR, our new structuring element showed overall better results in sequence 1–3, except for in a few frames (i.e., frames 17–22, sequences 2 and 3), and much better results (mostly 100% better) in sequences 4 and 5 for all frames, which indicates that our solution is superior for dealing with complex backgrounds. For BSF, our structuring element did yield better performances in sequences 1–3, but the differences were small (around 10%). In sequences 4 and 5, the performances were mixed, and no clear winner could be identified. In other words, there was no clear advantage of our specifically designed structuring element over the original element from NWTH when being used solely. However, our structuring element became more effective when combined with image decomposition in the proposed algorithm. As shown in Table 3 and Table 4, our combined approach achieved better scores in both SCR and BSF for all sequences.

## 5. Conclusions

In this paper, we presented a combined approach to detect small targets in infrared images which contained two key separate steps. The first was to convert small-target detection problems into optimization problems of low-rank sparse matrix recovery, where ADMM (without sliding steps) was used for preliminary background suppression. The second key step was to process the obtained interim images via an improved NWTH transformation with a specifically designed threefold structuring element, where the targets were further separated from the noise and clutter. The method was described in a workflow of four stages: (1) Image input; (2) image decomposition; (3) morphological filtering; and (4) thresholding segmentation. The outcomes of the workflow at each stage showed the effectiveness of the combined approach, which was conducted through four images with typical backgrounds (i.e., sky, cloud, land, and sea). In the evaluation against other state-of-art methods, our method showed superior results in both visual and quantitative comparisons. When compared to the baseline max-mean method, two top-hat-based methods (i.e., classical top-hat and NWTH), and two HVM-based methods (i.e., LCM and RLCM), our approach outperformed them in all SCR, BSF, and false alarm rate results. In addition, the effectiveness of the improved threefold structuring element against the one from NWTH was further demonstrated in the head-to-head comparisons of SCR and BSF. In addition, when compared to IPI, our method requires significantly less processing time and more consistent detection performances among the five different types of image sequences.

## Figures and Tables

**Table 1:** The properties of the image sequences. “*” means to converted to grey-scale images in the step “Image Input”.

Sequence	Target Region	Frames	Frame Size	Avg SCR	Image Description
Seq 1	5 × 5	30	256 × 200	0.62	Sky background; mostly covered by scattered clouds; fixed camera position and the target is from left to right.
Seq 2	5 × 5	30	238 × 158	0.65	Sky and sea background, with a clear horizontal boundary; fixed camera position and the target is moving from top to bottom.
Seq 3	5 × 5	30	302 × 202	0.51	Sky background; partially covered by thick cloud; fixed camera position and the target is moving from right to left.
Seq 4 *	4 × 4	30	256 × 256	3.81	Land background (Rapidly changing); tracking camera position; small target (16 pixels).
Seq 5 *	3 × 3	30	256 × 256	1.63	Land background; tracking camera position; ultra-small target (9 pixels).

**Table 2:** The parameters of the comparison methods.

Methods	Parameter Settings
Max-mean	Sliding window size=21 × 21
Top-hat	Structuring element size=5 × 5
NWTH	$R_{O}=9,\;R_{i}=4$$\;\mathrm{for}\;\mathrm{sequences}\;1\text{–}4;\;R_{O}=8,\;R_{i}=3$ for sequence 5
LCM	Cell size v=3, h = 3, 5, 7, 9
RLCM	$\mathrm{Scale}=3;\;k_{1}=2,\;5,\;9k_{2}=4,\;9,\;16$
IPI	Patch size=80 × 80 $,\;\mathrm{sliding}\;\mathrm{step}=5,\;\lambda =1/\sqrt{max\left(m,n\right)}$
Ours	$S_{o}=7,\;S_{i}=3$$\;\mathrm{for}\;\mathrm{sequences}\;1\text{–}4;\;S_{o}=5,\;S_{i}=2$ for sequence 5; other parameters are shown in Algorithm 1.

**Table 3:** Average SCR (notation: best result, second-best result).

Method	Sequence 1	Sequence 2	Sequence 3	Sequence 4	Sequence 5
Max-mean	10.48	26.91	4.10	9.67	14.87
Top-hat	6.77	6.89	24.96	7.28	10.27
NWTH	**79.46**	137.34	66.40	**22.52**	13.63
LCM	3.17	1.23	3.36	5.50	4.92
RLCM	65.23	136.16	**886.35**	14.49	12.72
IPI	21.24	**146.91**	142.91	9.95	**72.37**
Ours	**265.84**	**240.54**	**490.00**	**35.26**	**20.68**

**Table 4:** Average BSF (notation: best result, second-best result).

Method	Sequence 1	Sequence 2	Sequence 3	Sequence 4	Sequence 5
Max-mean	3.96	12.75	6.69	4.26	1.48
Top-hat	1.05	3.43	8.39	2.05	0.81
NWTH	5.65	13.20	20.07	11.92	3.29
LCM	0.92	0.93	0.95	1.54	0.54
RLCM	2.02	3.98	6.87	6.97	1.27
IPI	**10.96**	**14.41**	**22.92**	**17.76**	**3.99**
Ours	**7.17**	**14.15**	**23.69**	**14.09**	**3.98**

**Table 5:** Average processing time of a single frame (notation: best result, second-best result).

Method	Sequence 1	Sequence 2	Sequence 3	Sequence 4	Sequence 5	Average
NWTH	**0.015**	**0.014**	**0.016**	**0.017**	**0.016**	**0.016**
IPI	17.39	13.03	60.62	31.93	23.45	29.28
Ours	**1.32**	**0.77**	**2.60**	**3.70**	**2.86**	**2.25**

## Data Availability

The data presented in this study are available on request from the corresponding author.
